# Mitogen-Activated Protein Kinases SvPmk1 and SvMps1 Are Critical for Abiotic Stress Resistance, Development and Pathogenesis of *Sclerotiophoma versabilis*

**DOI:** 10.3390/jof9040455

**Published:** 2023-04-07

**Authors:** Felix Abah, Yunbo Kuang, Jules Biregeya, Yakubu Saddeeq Abubakar, Zuyun Ye, Zonghua Wang

**Affiliations:** 1 Ministerial and Provincial Joint Innovation Centre for Safety Production of Cross-Strait Crops, College of Life Sciences & College of Plant Protection, Fujian Agriculture and Forestry University, Fuzhou 350002, China; 2 The Engineering Technology Research Center of Characteristic Medicinal Plants of Fujian, College of Life Sciences, Ningde Normal University, Ningde 352100, China; 3 Fujian Provincial Key Laboratory on Conservation and Sustainable Utilization of Marine Biodiversity, Fuzhou Institute of Oceanography, Minjiang University, Fuzhou 350108, China

**Keywords:** SvPmk1, SvMps1, pathogenicity, *Sclerotiophoma versabilis*, vegetative growth, cell wall integrity

## Abstract

Mitogen-activated protein kinase (MAPK) signaling pathways are evolutionarily conserved in eukaryotes and modulate responses to both internal and external stimuli. Pmk1 and Mps MAPK pathways regulate stress tolerance, vegetative growth and cell wall integrity in *Saccharomyces cerevisiae* and *Pyricularia oryzae*. Here, we deployed genetic and cell biology strategies to investigate the roles of the orthologs of Pmk1 and Mps1 in *Sclerotiophoma versabilis* (herein referred to as SvPmk1 and SvMps1, respectively). Our results showed that SvPmk1 and SvMps1 are involved in hyphal development, asexual reproduction and pathogenesis in *S. versabilis*. We found that *∆Svpmk1* and *∆Svmps1* mutants have significantly reduced vegetative growths on PDA supplemented with osmotic stress-inducing agents, compared to the wild type, with *∆Svpmps1* being hypersensitive to hydrogen peroxide. The two mutants failed to produce pycnidia and have reduced pathogenicity on *Pseudostellaria heterophylla*. Unlike SvPmk1, SvMps1 was found to be indispensable for the fungal cell wall integrity. Confocal microscopic analyses revealed that *SvPmk1* and *SvMps1* are ubiquitously expressed in the cytosol and nucleus. Taken together, we demonstrate here that SvPmk1 and SvMps1 play critical roles in the stress resistance, development and pathogenesis of *S. versabilis*.

## 1. Introduction

*Pseudostellaria heterophylla* is a Chinese medicinal plant. It contributes significantly to the Traditional Chinese Medicine practices and local economy of eastern Fujian. *P. heterophylla* was domesticated because of its medicinal and economic importance [1,2]. However, the plant is faced with leaf spot disease, a devastating fungal disease caused by *Sclerotiophoma versabilis* [3]. It has caused an enormous loss of quality and yield and reduced this important herbal plant’s medicinal and economic value. The loss to this fungal disease ranges from 15% to 50% every year [4,5]. Understanding the mechanism of *S. versabilis* pathogenesis at the molecular level helps to explore the potential targets of pharmaceutical molecules and provide new measures for the control of *P. heterophylla* leaf spot disease.

Mitogen-activated protein kinase (MAPK) signaling pathways consist of Serine/Threonine kinases [6] and are conserved in all eukaryotes [7]. MAPK pathways play important roles in the regulation of different developmental processes as well as in the response to host and environmental signals in fungal pathogens [8]. The regulation is through the dual phosphorylation of threonine and tyrosine residues of the conserved TXY motif within the activation loop by MEKK, MEK and MAPK [8,9,10]. In *Saccharomyces cerevisiae*, five MAPK pathways (Kss1, Fus3, Hog1, Slt2 and Smk1) are known to regulate mating, invasive growth, cell wall integrity, hyper-osmoregulation and ascospore formation [11]. The MAPK signaling pathways of plant pathogenic fungi are significantly different from those of *S. cerevisiae*. In plant pathogenic fungi, Fus3 and Kss1 pathways have partial functional redundancy, so Fus3 and Kss1 pathways are classified into the same category, while the Smk1 signaling pathway has not been found in plant pathogenic fungi [12,13]. Therefore, the MAPK signaling pathway of plant pathogenic fungi can be divided into three categories: the Fus3/Kss1 pathway is mainly involved in regulating the mating response of pathogenic fungi, and the formation of appressorium, mycelium infection and disease. The Slt2 pathway is a cascade involved in cell wall integrity. The Hog1 pathway is responsible for the response to high osmolarity [8].

Pmk1, Mps1 and Osm1 (orthologs of Fus3/Kss1, Slt2 and Hog1, respectively) have been studied well in numerous pathogenic fungi [13,14,15,16]. Although the Pmk1, Mps1 and Osm1 MAPK pathways contribute to fungal virulence, they have distinct functions in other developmental processes, and these functions vary among fungal species [8,17].

Pmk1 (ortholog of Fus3/Kss1) controls the septin-dependent morphogenesis of invasive hyphae and plays a significant role in regulating hyphal constriction to transverse pit fields of the host tissue [16,18]. In appressorium-forming fungal pathogens (such as *Pyricularia oryzae*), the Pmk1 protein is directly involved in appressorium formation, host penetration and invasive growth [17,19,20]. The pathway regulates the induction of appressorium formation after the fungus senses a natural or artificial hydrophobic surface [10,21]. For other pathogens that do not form appressorium, such as *Mycosphaerella graminicola*, it plays a very important role in the process of invasion, expansion and asexual reproduction [22,23,24]. Mps1 not only affects the infection and cell wall integrity of *P. oryzae*, but also involves other biological processes such as conidium production and sclerotium formation [8].

According to Li et al. [25], Mps1 is unnecessary for appressorium formation in *P. oryzae* and other appressorium-forming fungi. However, it is indispensable for cell wall integrity, appressorium-mediated penetration, invasive growth, transpressorium [26,27] and cell wall construction and maintenance during intracellular and extracellular changes [28]. In addition, Mehrabi et al. [29] demonstrated that the germ tube of the *Mgslt2* mutant strain of *Mycosphaerella graminicola* could penetrate wheat stomata; however, it could not branch into invasive hyphal growth to cross to the adjacent cells. More so, Mps1 regulates the accumulation of alpha-1, 3-glucan, provides protection against chitinases and suppresses immunity during plant infection [25].

Although the pivotal roles played by Mps1 and Pmk1 were established in some model organisms such as *S. cerevisiae and P. oryzae*, there is no known function of the MAPKs in the pathophysiological development of the economically important phytopathogen *Sclerotiophoma versabilis.* In this work, we investigated the functions of these two genes in *S. versabilis* in relation to pathogenicity, pycnidia and spore formations, cell wall integrity and general host–fungus interaction. We showed that the knockout mutants of the *SvPMK1* and *SvMPS1* genes restricted the growth of the *S. versabilis* infection of adjacent host cells. Our findings will be of broad interest to microbiologists and molecular plant pathologists.

## 2. Materials and Methods

### 2.1. Plants, Fungal Strain and Culture Condition

The wild type (WT) strain of *S. versabilis* KC1 (isolated from its host plant in Ningde, Fujian, China) was used to generate the mutants, and susceptible *P. heterophylla* plantlets were used for the plant infection assay. All the fungal strains were cultured on potato dextrose agar [PDA: 40.1 g PDA powder in 1 L double-distilled water (ddH_2_O)] (Guangdong Huankai microbial and Tech. Co., Ltd., Guangzhou, China) at 25 °C as described previously [30] with little modifications. Other media used included oatmeal agar (OA: 40 g of oatmeal granules and 20 g of agar powder in 1 L of ddH_2_O), malt extract agar (MEA: 20 g malt extract powder and 15 g of agar powder in 1 L of ddH_2_O), potato dextrose broth (PDB: 24 g PDB powder in 1 L ddH_2_O) (Guangdong Huankai microbial and Tech. Co., Ltd., Guangzhou, China) and TB3 (6 g Casamino acid, 6 g of yeast extract, 200 g of sucrose and 20 g of agar in 1 L of ddH_2_O). For protoplast isolation and genomic DNA extraction, samples were grown in PDB and liquid complete medium (CM: 6 g yeast extract, 6 g casein hydrolysate and 10 g sucrose in 1 L ddH_2_O) [31] in an orbital shaker at 25 °C and 110 rpm for 3 to 5 days. To induce conidiation, strains were cultured on the host leaf agar (HLA) media (20 g agar in 1 L of ddH_2_O, 4 pieces of sterile leaves from tissue culture).

The plasmids pCX64 (for gene knockout) and pKNTG (for complementation) used in this study were sourced from Dr. Justice Norvienyeku of the State Key Laboratory for Plant-Microbe Interaction, Plant Protection College, Fujian Agriculture and Forestry University, Fuzhou, China. The vectors contained hygromycin and Geneticin resistance genes, respectively. The *Escherichia coli* strain (*DH5α*) was used for bacterial transformation.

For sensitivity assays, each strain was cultured on PDA supplemented with cell wall stressors [0.01% SDS (Sinopharm Chemical Reagent Co., Ltd., Shandong, China), 200 µg/mL Calcofluor white (Sigma−Aldrich Co., Missouri, USA), 200 µg/mL Congo red (Hefei Bomei Biotechnology Co., Ltd., Hefei, China); osmoticum [1 M NaCl, 1 M KCl, 1 M sorbitol (Beijing Solarbio Sci. and Tech., Beijing, China)] and oxidative stressor 2.5 mM and 5 mM hydrogen peroxide, H_2_O_2_ (Sinopharm Chemical Reagent Co., Ltd., Shandong, China) for seven days at 25 °C in a 12/12 h dark/light photoperiod. Each experiment had five replicates and was repeated three times.

### 2.2. Generation of Gene Deletion Mutants

Protoplast preparation and PEG−mediated transformation were performed as protocols as previously described [32] with some modifications. The purified protoplasts were resuspended in 3 mL 1× STC sorbitol and estimated using a hemocytometer (XB. K. 25. Improve Neubauer China). There were five replicates for every treatment; the experiment was conducted three times.

Homologous recombination was used to obtain *SvPmk1* and *SvMps1* mutants (Supplementary Figure S1). Based on the conservation of eukaryotic MAPK proteins and the whole genome database of *S. versabilis* (GenBank accession number: AJAAROD000000000, BioProject: PRJNA613783), the homologous alignment method was used to find out *SvPmk1* and *SvMps1* orthologous to the key genes *Pmk1* and *Mps1* in the MAPK signaling pathway of *P. oryzae*, respectively. The upstream (A) and downstream (B) flanking regions of *SvPmk1* and *SvMps1* were amplified by PCR. The forward primers were designed to introduce *EcoR*I site at the 5′ ends, and the reverse primers were designed to introduce *BamH*I site at the 3′ ends. The A fragments were ligated with the upstream half of hph on pCX62, and the B fragments were ligated with the downstream half of hph on the pCX62 (Bryksin and Matsumura 2013). AH and BH for the *SvPmk1* and *SvMps1* gene knockout were separately amplified by PCR using each recombinant DNA as the template. Knockout candidates were confirmed by PCR and Southern blotting. At least three transformants were obtained for each gene. Primers used for flanking sequences’ amplification, AH and BH amplification and mutants’ PCR screening for each gene are listed in Supplementary Table S1.

### 2.3. Mutant Complementation

For complementation of the *SvPMK1* and *SvMPS1* mutants, the entire length of *SvPMK1* (2805 bp) and *SvMPS1* (4554 bp) genes including the ORF and their respective native promoter sequences were amplified by PCR from the wild type strain. The purified target band was ligated to the pKNTG-GFP vector. The constructed vector was transformed into the *SvPMK1* and *SvMPS1* mutants, respectively. Then, transformants were confirmed by PCR and Southern blotting. Primers used for complementation DNA fragment amplification and mutants’ PCR screening for each gene are listed in Supplementary Table S1.

### 2.4. DNA Extraction, Gel Electrophoresis and Southern Blot Analysis

Total genomic DNA was extracted from liquid nitrogen−frozen mycelia of the WT, *∆Svpmk*, *∆Svmps1* and complemented strains using cetyltrimethylammonium bromide (CTAB) [31] or the phenol−chloroform (PCI) DNA extraction method [33]. The mycelia samples cultured in PDB were incubated in an orbital shaker for five days at 25 °C, 110 rpm. Mycelia were then filtered out, rinsed with ddH_2_O, dried with sterile absorbent filter paper (NEWSTAR, Hanzhou, China), frozen and ground in liquid nitrogen. About 0.5 g of the samples was placed in 2 mL Eppendorf centrifuge tubes containing 0.5 mL DNA extraction buffer 1 (100 mM Tris-HCl pH 8.0, 100 mM EDTA and 250 mM NaCl) and vortexed. About 0.05 mL of 20% SDS was added and incubated at 37 °C for 1 h, shaking every 20 min. Then, 0.075 mL of 5 M NaCl and 0.065 of mL 0.75 M NaCl + 10% CTAB were added, mixed gently and incubated at 65 °C for 20 min. After cooling, 0.05 mL of Chloroform:Tri-phenol (1:1 *v*/*v*) was added, mixed thoroughly for 10 min and centrifuged for 10 min at 12,000 rpm. The supernatants were pipetted into new Eppendorf tubes containing an equal volume of chloroform, mixed for 5 min and centrifuged at 12,000 rpm. The supernatant was collected into new 2 mL Eppendorf tubes containing X2 volumes of chilled absolute ethanol and incubated at −0 °C overnight for DNA precipitation. The mixtures were centrifuged for 10 min at 12,000 rpm, and the resulting supernatants were discarded. The DNA pellets were oven-dried at 65 °C for 10 min. The dried DNA pellets were dissolved in 0.5 mL ddH_2_O containing 2 µL RNase A and incubated at 37 °C for 30 min. Afterwards, an equal volume of chloroform:isoamyl alcohol (24:1 *v*/*v*) was added, mixed thoroughly for 10 min and centrifuged for 10 min at 12,000 rpm. The supernatants were transferred into sterile 1.5 mL Eppendorf tubes, and a two-fold volume of chilled absolute ethanol was added and incubated for 2 h at −20 °C. The samples were centrifuged at 12,000× *g* for 10 min, and the supernatants were discarded. The DNA pellets were washed with 1 mL 70% ethanol and oven-dried. The dried DNA pellets were dissolved in 50 µL of sterile ddH_2_O and used for Southern blotting and other experiments.

Gel electrophoresis, enzymatic DNA digestion and purification, ligation and Southern blot hybridization were carried out according to the procedures described previously [34,35,36,37,38] with minor modifications. Probing, hybridization, staining and balance were performed using the DIG HIGH Prime DNA Labelling and Detection Starter Kit I (Roche Diagnostics GmbH, Mannheim, Germany).

To digest DNA, a total volume of 100 µL of reaction mixture consisting of 62 µL sterile ddH_2_O, 10 µL 10× FastDigest buffer, 20 µL genomic DNA (2000 ng/µL) and 8 µL *Hind*III (*Kpn*1 for SvMPS1) restriction enzymes was incubated for 24 h and gel electrophoresed to confirm the digestion. Afterwards, two-fold of ethanol absolute was added to the digested DNA and incubated at −20 °C for about 2 h. Then, the mixture was centrifuged at 12,000× *g* rpm for 10 min and the supernate was discarded. Next, 1000 µL 70% ethanol was added and centrifuged at 12,000× *g* for 2 min to wash the DNA pellet. The supernatant was discarded, and the DNA pellet was dried in the laminar air-flow chamber for 20–30 min. The dried DNA was suspended in 40 µL of sterile ddH2O and saved at −20 °C. Restriction enzymes were found using PimerPrimer5 application software.

For probe preparation, the reaction mix was prepared as follows: 5 µL of sterile ddH_2_O, 10 µL A fragment DNA of SvPMK1 (B fragment for SvMPS1) and 1% (*v*/*v*) marker IV were added in a microcentrifuge tube and boiled for 10 min. Then, 4 µL solution I (DIG High Prime DNA) was added and incubated at 37 °C for 24 h. Then, the probe was set at 65 °C in a water bath for 10 min to stop the reaction before being used for probing. A fragment (upstream) of SvPMK1 and SvMPS1, respectively, used in probing were amplified using primer pairs Pmk-AF/AR for *SvPMK1* and Mps1-AF/AR for SvMPS1.

### 2.5. Plant Penetration Assay

Host penetration and invasive hyphal growth assays were performed by inoculating the underside of wounded detached leaves of susceptible *H. heterophylla*. The penetration assay was also conducted on both 7-day-old injured barley leaves (golden promise cultivar) and onion epidermis with mycelial plugs from the 5-day-old culture of WT, *∆Svpmk1*, *∆Svmps1* and complemented strains. The cultures were incubated in the dark at 25 °C in a humid condition. Disease progression and mycelial penetration were then examined under a light microscope after 8, 12 and 24 h as described previously (Lin et al. 2021) with slight modifications.

### 2.6. Pathogenicity Assay

For the pathogenicity assay, a hyphae-mediated infection was conducted by inoculating the host leaf with 5-day-old mycelia of the WT, mutants and complemented strains. Each strain was pre-cultured in PDA for five days at 25 °C. The 5-day-old mycelial plugs from WT, *∆Svpmk1*, *∆Svmps1* and complemented strains were used for inoculation on detached wounded host leaves. The infected leaves were then incubated at 25 °C, 45% RH for seven days in the diurnal light to mimic the day and night natural photoperiod. Disease development on the leaves was imaged after seven days.

### 2.7. Microscopic Examination

An Olympus DP80 light microscope (Tokyo, Japan) was used to observe the hyphal penetration and invasion on media surfaces. GFP localization was observed using a laser confocal microscope equipped with a Nikon A1 Plus imaging instrument (Tokyo, Japan).

### 2.8. Growth Assay

For the growth rate assay, *S. versabilis* WT, *∆Svpmk1*, *∆Svmps1* and complemented strains were inoculated on PDA media and incubated at 25 °C, 45% RH for seven days in the 12 h light/12 h dark photoperiod. The diameter of each colony was then measured in two perpendicular directions, and an average of the two measurements was calculated after subtracting the 5 mm diameter of the colonized plug [30]. There were five replicates of Petri dish plates per strain, and the experiment was repeated three times.

## 3. Results

### 3.1. Identification of SvPmk1 and SvMps1 in S. versabilis

**Figure 1:**
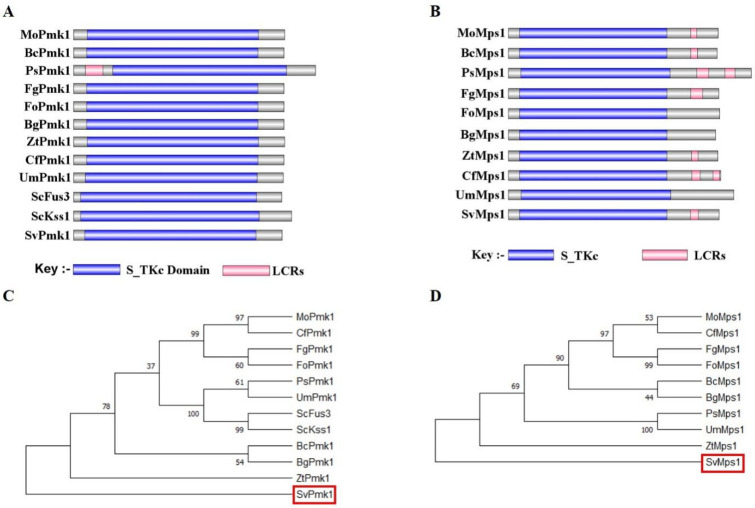
Domain architecture and phylogeny of Pmk1 and Mps1 in different fungi. *S. cerevisiae* Kss1 (NP_011554), *S. cerevisiae* Fus3 (AAA34613.1), *M. oryzae* Pmk1 (XP_003712175), *Botrytis cinerea* Pmk1 (XP_024547333.1), *Puccinia striiformis* Pmk1 (KNE97907.1), *Fusarium graminearum* Pmk1 (XP_011325047.1), *F. oxysporum* Pmk1 (XP_018244117.1), *Blumeria graminis* Pmk1 (AAG53654.2), *Zymoseptoria tritici* Pmk1 (XP_003851863.1), *Colletotrichum fructicola* Pmk1 (XP_031881962.1), *Ustilago maydis* Pmk1 (XP_011389711.1). *M. oryzae* Mps1 (XP_003712437), *Botrytis cinerea* Mps1 (XP_001554555.1), *Puccinia striiformis* Mps1 (KNE99304.1), *Fusarium graminearum* Mps1 (P_011319273.1), *F. oxysporum* Mps1 (XP_018240084.1), *Blumeria graminis* Mps1 (CAD6500573.1 BgTH12-06283), *Zymoseptoria tritici* Mps1 (XP_003856015.1), *Colletotrichum fructicola* Mps1 (XP_031889253.1), *Ustilago maydis* Mps1 (XP_011386664.1). (**A**,**C**) Pmk1 domain architecture and phylogenetic analysis in different fungi. (**B**,**D**) Mps1 domain architecture and phylogeny in different fungi. The evolutionary history was inferred using the maximum likelihood method based on the JTT matrix-based model. The analysis involved 22 amino acid sequences. The maximum likelihood phylogeny for the amino acids was tested with 1000 bootstrap replicates. Evolutionary analyses were conducted using MEGA7. LCRs: low complexity regions.

Amino acid sequences of Pmk1 and Mps1 from *P. oryzae* were used to conduct a BLASTp search at the FungiDB (https://fungidb.org, accessed on 12 August 2020) [39] and National Centre for Biotechnology Information (https://www.ncbi.nlm.nih.gov/, accessed on 12 August 2020) databases. Homologs of Pmk1 and Mps1 were subsequently identified in the *S. versabilis* genome and were named SvPmk1 and SvMps1, respectively. More homologs of these proteins were found in the other important fungi, which showed 71.29% sequence identities for SvPmk1 and 74.04% for SvMps1, and further analyzed at SMARTdatabase (http://smart.embl-heidelberg.de/, accessed on 12 August 2020) to identify and classify the various domains in the proteins [40]. The results showed that SvPmk1 and SvMps1 contain serine/thyronine protein kinase catalytic domain S_TKc which is conserved in all the top nine important plant pathogenic fungi (Figure 1A,B). A phylogenetic analysis indicated that SvPmk1 and SvMps1 have distant ancestry with Pmk1 and Mps1 of all the fungi analyzed (Figure 1C,D).

To determine the functions of SvPmk1 and SvMps1 in *S. versabilis*, we generated their respective deletion mutants by replacing the entire *SvPMK1* and *SvMPS1* genes from wild type (WT) with a hygromycin resistance gene using a homologous recombination approach (Supplementary Figure S1). The deletion mutants of the two genes were then confirmed by a PCR assay and Southern blot analysis (Supplementary Figure S2).

### 3.2. SvPmk1 and SvMps1 Are Essential for Vegetative Growth and Asexual Reproduction in S. versabilis

**Figure 2:**
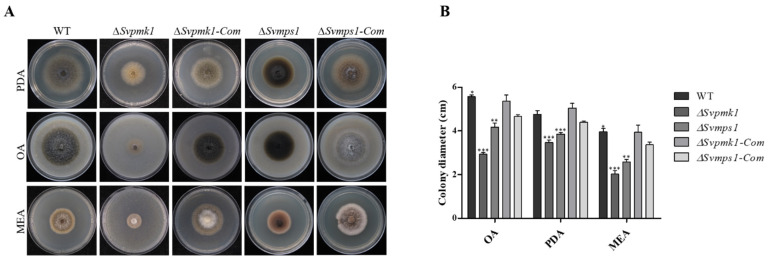
Roles of SvPmk1 and SvMps1 in the vegetative growth of *S. versabilis. (***A**) Vegetative growth of the WT, mutants and complemented strains on PDA, OA and MEA media at 7 days post inoculation (dpi). (**B**) Analysis of the colony diameters of the various strains at 7 dpi. Error bar = standard deviation from mean of three independent replicates; * p < 0.1; ** *p* < 0.01; *** *p* < 0.001.

To investigate the role of SvPmk1 and SvMps1 in the vegetative growth of *S. versabilis*, we grew the two mutants and the wild type (WT) on potato dextrose agar (PDA), oatmeal agar (OA) and malt extract agar (MEA) media and measured their colony diameters after 7 days of incubation. We observed significant differences in the colony diameters of the mutants relative to the WT and complemented strains (Figure 2A,B). In addition, the two mutant strains failed to develop aerial mycelia and pycnidia (asexual fruiting body of *S. versabilis*). The failure of the two mutant strains to produce pycnidial indicates that the *SvPMK1* and *SvMPS1* genes are essential for sporulation and asexual reproduction in *S. versabilis*. Additionally, the *∆SvMps1* mutant appeared highly melanized, suggesting that the *SvMPS1* gene could be a negative regulator of melanin biosynthesis (Figure 2A). The reintroduction of the *SvPMK1* and *SvMPS1* genes into the mutant strains restored normal growth and pigmentation on the PDA, OA and MEA (Figure 2A). This result shows that SvPmk1 and SvMps1 are needed for the vegetative growth of *S. versabilis*.

### 3.3. SvPmk1, but Not SvMps1, Contributes to Osmotic Stress Resistance in S. versabilis

**Figure 3:**
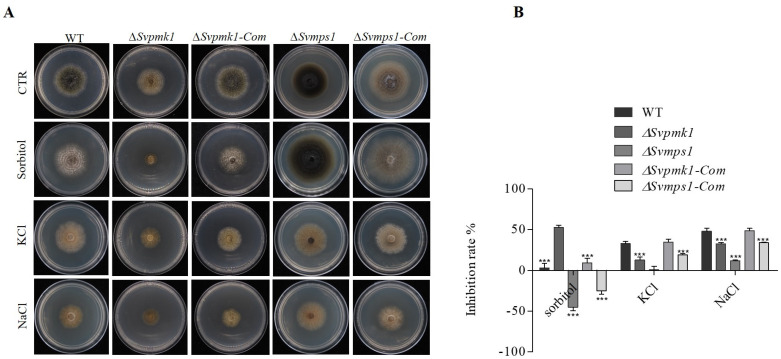
Roles of SvPmk1 and SvMps1 in osmotic stress tolerance in *S. versabilis*. (**A**) The WT, *∆Svpmk1*, *∆Svmps1*, *∆Svpmk1-com* and *Svmps1-com* were cultured on PDA supplemented with 1 M Sorbitol, KCl and NaCl and incubated at 25 °C for 7 days for osmotic stress response. (**B**) Percentage inhibition of vegetative growth of the mutants relative to the WT and complementation strains. The inhibition rate of each treatment was compared with the growth rate of the untreated control. Inhibition rate = (the colony diameter of untreated strain−the colony diameter of the treated strain)/(the colony diameter of untreated strain) × 100. Error bar represents SD from mean value of five replicates. Asterisk = *p*-value (***, *p* < 0.001). Statistical analyses were conducted using a two-way analysis of variance (ANOVA) in GraphPad Prism 5 and Microsoft Excel spreadsheets. Similar values were obtained from three independent experimental repeats with five technical replicates for each repetition.

In fission yeast, Pmk1 positively regulated Na^+^ tolerance but increased the sensitivity of the fungus to K^+^ [41]. Similarly, *Cmekk1* and *Cmk1* (homologs of Pmk1 and Mps1, respectively) mutants of *Colletotrichum orbiculare* were highly sensitive to salt stress [42]. For these reasons, we decided to investigate the role of SvPmk1 and SvMps1 in response to osmotic stress tolerance in *S. versabilis*. To achieve this, the WT, *∆Svpmk1*, *∆Svmps1* and the complemented strains were cultured on PDA media supplemented with 1 M NaCl, KCl and C_6_H_14_O_6_ (sorbitol) as osmotic stress-inducing agents and their respective tolerance evaluated. The results obtained showed that the *∆Svpmk1* mutant strain was more inhibited by NaCl, KCl and C_6_H_14_O_6_ than the *∆Svmps1 strain*_._ Although the WT and complemented strains exhibited more sensitivity to NaCl and KCl, they were less sensitive in the C_6_H_14_O_6_-supplemented medium compared to the *∆Svpmk1* mutant (Figure 3A,B). In contrast, we observed that *∆Svmps1* was tolerant to all the salts, and it was less sensitive than the other strains tested. Furthermore, the vegetative growth of the SvMps1-deficient strain exceeded that of the WT and complemented strains. We, therefore, conclude that SvPmk1 (but not SvMps1) contributes to osmotic stress tolerance in *S. versabilis*.

### 3.4. SvMps1 Is Required for Cell Wall Integrity Maintenance in S. versabilis

**Figure 4:**
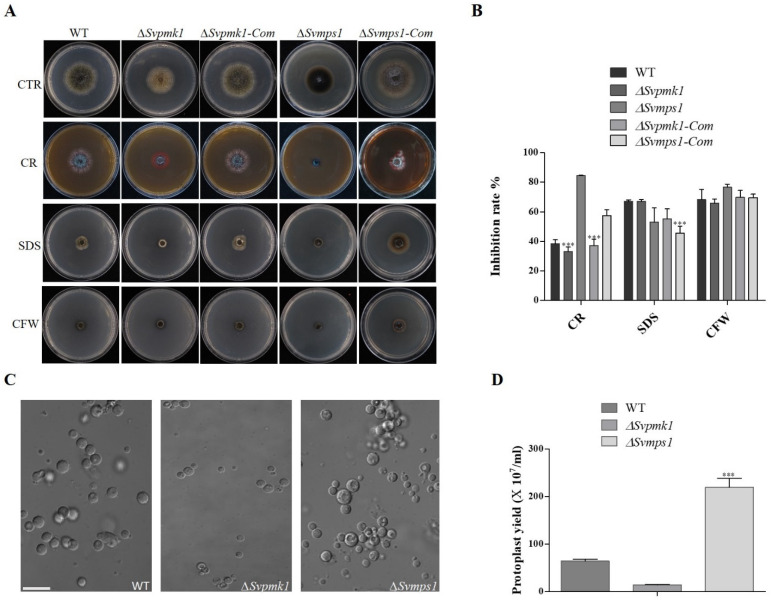
Sensitivity of *∆Svpmk1*, *∆Svmps1* and their complemented strains to cell wall stressors. (**A**) Vegetative growth of the WT, mutant and complemented strains cultured on PDA supplemented with the cell wall stressors (200 μg/mL CR, 0.01% SDS and 200 μg/mL CFW) and imaged 7 dpi. (**B**) Percentage growth inhibition of the WT, *∆Svpmk1*, *∆Svmps1* and complemented strains on CR, SDS and CFW. (**C**) Protoplasts from the WT, *∆Svpmk1* and *∆SvMps1* after treatment with lysing enzyme at 30 °C 85 rpm for 2 h. Error bar = 10 µm. (**D**) Amount of protoplast released by the WT and the two mutant strains. The inhibition rate of each treatment was compared with the growth rate of the untreated control. Inhibition rate = (the colony diameter of untreated strain—the colony diameter of the treated strain)/the colony diameter of untreated strain) × 100. Asterisk = *p*-value ***, *p* < 0.0001). Statistical analysis was conducted using two-way ANOVA with Tukey’s multiple-comparison test (GraphPad Prism 5). Error bar represents standard deviation from the mean. The experiments were conducted three times with five independent replicates.

Every external stress signal needs to overtake the cell wall before an appropriate stress response [43]. To investigate the contribution of the SvPmk1 and SvMps1 MAPKs to cell wall stress resistance in *S. versabilis*, we cultured the various fungal strains on PDA media supplemented with the cell wall stressors calcofluor white (CFW), sodium dodecyl sulphate (SDS) and Congo red (CR) [20,31,44]. After 7 days of inoculation, we analyzed the colony diameters of the cultures. Our results showed that the vegetative growth of the *∆Svmps1* mutant was highly inhibited (followed by the *∆Svpmk1* mutant) on CR-supplemented media, as compared to the control cultures (Figure 4). Both the WT and *∆Svpmk1* mutant were similarly inhibited on the SDS-containing medium, with the highest rate recorded in *∆Svmps1*. On the CFW medium, however, there were nearly zero vegetative growths among all the strains tested (Figure 4A,B).

To further confirm the role of SvMps1 in cell wall integrity, we exposed the mycelia of the strains under study to a cell wall-degrading enzyme (25 mg/mL lysing enzyme) and measured the number of protoplasts formed within a given period. Our results here showed that the *∆Svmps1* mutant had the highest number of protoplasts, followed by the WT control and then the *∆Svpmk1* mutant within 2 h of swirling at 85 rpm and 30 °C (Figure 4C), indicating that the cell wall of the *∆Svmps1* mutant was more prone to degradation than those of the other strains.

### 3.5. SvMps1 Is Required for Tolerance to Oxidative Stress

**Figure 5:**
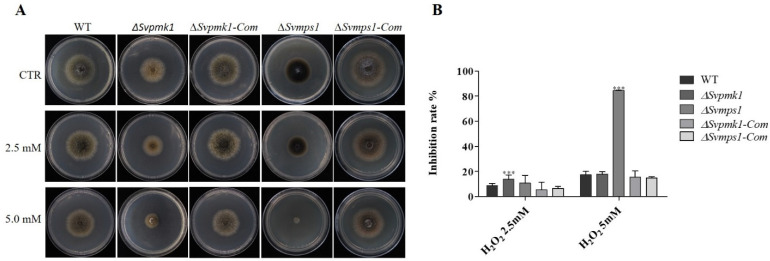
Sensitivity of *∆Svpmk1* and *∆Svmps1* to different concentrations of H_2_O_2_**.** (**A**) Growth phenotype of the WT, mutants and complemented strains under oxidative stress condition. The WT, mutant and complemented strains were cultured on PDA media supplemented with 2.5 and 5.0 mM H_2_O_2_, incubated at 28 °C for 7 days and then sampled for sensitivity assay. CTR was a full-strength PDA without H_2_O_2_. (**B**) The growth inhibition rate of the WT, mutants and complementation strains. Asterisk = *p*-value (***, *p* < 0.001). Error bar represents the standard deviation from the mean of three independent repeats with five technical replicates. Statistical analysis was conducted using two-way ANOVA with Tukey’s multiple-comparison test (GraphPad Prism 5).

The oxidative stress response protects organisms from the deleterious effects of reactive oxygen species (ROS) [45]. Hydrogen peroxide (H_2_O_2_) is one of the essentially toxic reactive oxygen species generated by innate immune cells as a fungicidal defense mechanism [46]. In *S. cerevisiae*, *Aspergillus niger* and other filamentous fungi, the use of H_2_O_2_ for the sensitivity assay intra- and extracellularly is well documented [47]. To investigate the contribution of SvPmk1 and SvMps1 proteins to oxidative stress tolerance in *S. versabilis*, we cultured the studied strains on PDA media supplemented with 0.25 mM or 5 mM H_2_O_2_ and analyzed the fungal vegetative growths at 7 dpi. We discovered that the growth of the *∆Svmps1* mutant was completely inhibited on media containing 5 mM H_2_O_2_ (Figure 5). According to the results, the *∆Svpmk1* mutant was more sensitive to both 2.5 and 5.0 mM H_2_O_2_ (Figure 5A,B) relative to the WT. We, therefore, conclude that the *SvMPS1* gene is required for tolerance to oxidative stress conditions in *S. versabilis*.

### 3.6. SvPmk1 and SvMps1 Are Important for Full Virulence of S. versabilis

**Figure 6:**
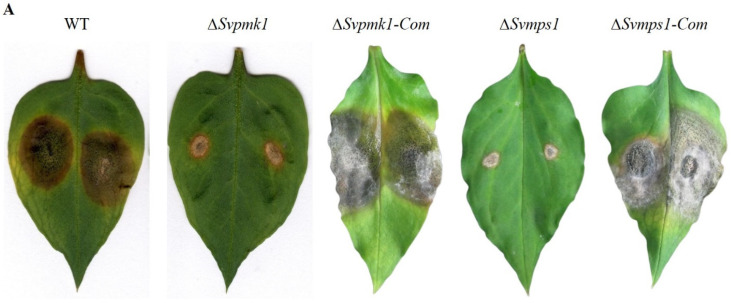

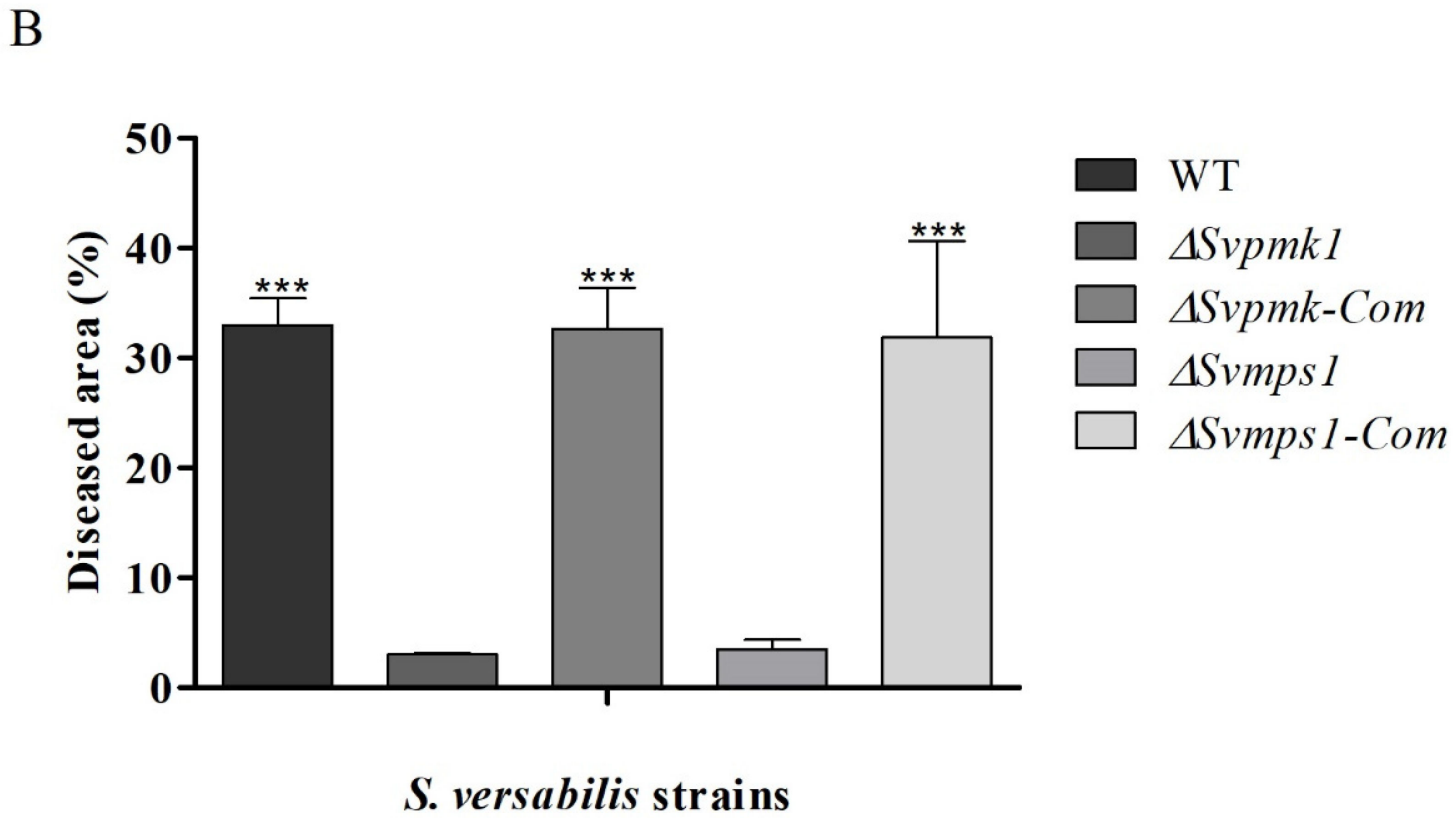
Roles of SvPmk1 and SvMps1 on pathogenicity of *S. versabilis*. (**A**) Disease symptoms on wounded detached leaves of *P. heterophylla* inoculated with mycelial plugs from WT, *∆Svpmk1*, *∆Svpmk1-com*, *∆Svmps1* and *∆Svmps1-com* strains in that order. *∆Svpmk1* and *∆Svmps1* failed to develop serious disease lesions on *P*. *heterophylla* wounded leaves. Typical leaves were photographed 14 days after inoculation. (**B**) Percentage of diseased area on the WT, mutants and complementation strains. Asterisk = *p*-value (***, *p* < 0.0005). Error bar represents the standard deviation from the mean of three independent repeats. Statistical analysis was conducted using ImageJ software and one-way ANOVA with Tukey’s multiple-comparison test (GraphPad Prism 5).

To establish the roles of SvPmk1 and SvMps1in the pathogenicity of *S. versabilis*, we inoculated 3-week-old host leaves with mycelial plugs from the WT, *∆Svpmk1*, *∆Svmps1*, *Svpmk-Com* and *Svmps1-com*, and incubated for 7 days at 25 °C. After this period, we found that the two mutant strains *∆Svpmk1* and *∆Svmps1* failed to cause serious disease symptoms on the susceptible host leaves (Figure 6). Only similar small lesions that could not expand after one to several weeks were observed around the points of inoculation of the mutants, compared to the wild type and complemented strains. On the other hand, the WT, *∆Svpmk1-Com* and *∆Svmps1-Com* strains produced similar large lesions on the leaves with numerous brown spherical fruiting bodies (pycnidia) typical of *S. versabilis* (Figure 6). Based on these results, we conclude here that SvPmk1 and SvMps1 are necessary for the full virulence of *S. versabilis*.

**Figure 7:**
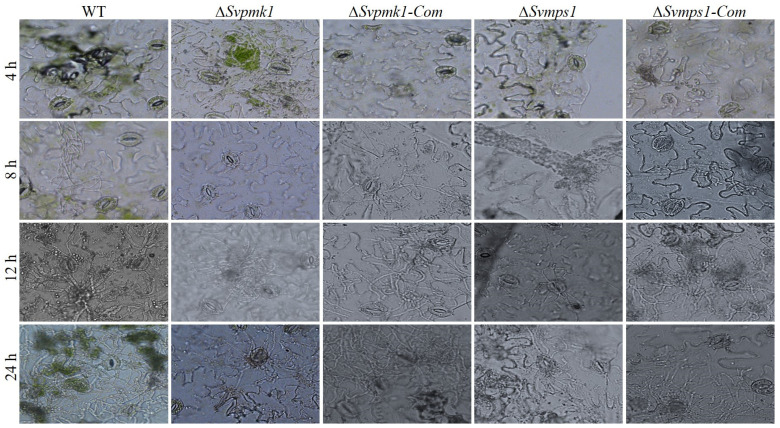
Penetration assay of the WT and the indicated strains of *S. versabilis*. Mycelial plugs from 5-day-old cultures were inoculated on 3-week-old host leaves. The hyphal growths were observed at 4, 8, 12 and 24 h. Scale bar = 50 μm.

Considering the above results, we hypothesized that the observed weak pathogenicity of the mutants was due to delayed penetration through the host cuticle into the surrounding tissues. To test this hypothesis, we inoculated physically injured host leaves with mycelial plugs from the WT, *∆Svpmk1*, *∆Svmps1* and complemented strains. We monitored the hyphal penetration and invasive growth after 4, 8, 12 and 24 h post-inoculation (hpi). We discovered that the WT, *∆Svmps1* mutant and complemented strains penetrated through the leaf cuticle after 8 hpi (Figure 7). After 12 h, the WT and complementation strains had invaded and colonized the adjacent cells of the host leaves (Figure 7) unlike the mutant strains whose hyphae were restricted to the point of inoculation. After 24 h, the *∆Svmps1* and Svpmk1 mutants penetrated the adjacent cell of the host leaves but failed to branch into invasive hyphae.

### 3.7. SvPmk1 and SvMps1 Are Localized to the Cytoplasm and Nucleus

**Figure 8:**
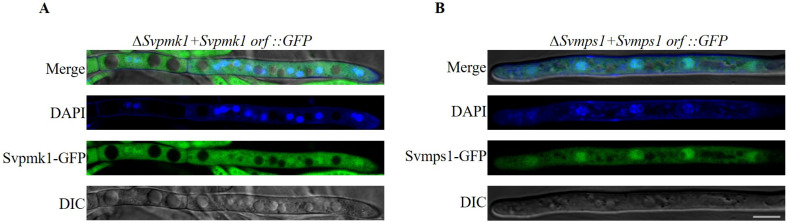
Subcellular localizations of SvPmk1-GFP and SvMps1-GFP. (**A**) SvPmk1-GFP is expressed throughout the cytoplasm and the nucleus. (**B**) SvMps1-GFP is co-localized with DAPI signal in the nuclei of the fungal mycelia. The mycelia were cultured in PDB at 25 °C, 180 rpm for 18 h. Scale bar = 20 µm.

To investigate the subcellular localizations of the SvPmk1 and SvMps1 proteins in *S. versabilis*, we fused *SvPMK1* and *SvMPS1* genes along with their respective native promoters with a GFP sequence in a pKNTG-GFP plasmid-containing geneticin resistant gene. The vectors were transformed into their respective mutant protoplasts and selected on TB3 solid media amended with geneticin. Fluorescence microscopy showed that SvPmk1 and SvMps1 were localized in the cytoplasm and nucleus (Figure 8A,B) and appeared as punctate structures in some given organelles. To ascertain the actual organelle in which the proteins were expressed, we stained the fungal hyphae with DAPI, a dye that clearly labels cell nuclei. We found a clear localization of the DAPI signals, suggesting that the SvPmk1 and SvMps1 proteins are localized at both the cytoplasm and the nucleus, although they are more concentrated at the nucleus (Figure 8).

## 4. Discussion

The mitogen-activated protein kinase (MAPK) signaling pathways are conserved in all eukaryotic organisms. The pathways are involved in internal and external environmental adaptive responses of the eukaryotic cells, thus playing an essential role in gene expression, mRNA stabilization and translation, cell cycle progression, cell proliferation and differentiation and cell survival. MAPK is activated by the dual phosphorylation of threonine and tyrosine residues [8]. The components of the signaling cascades are well-characterized in *Caenorhabditis elegans* [48], *S. cerevisiae* [8], *M. oryzae* [49] and some other filamentous fungi. However, their functions in *S. versabilis* remain unknown. Our study is interested in the genetic determinants of the filamentation and pathogenicity of *S. versabilis*. Here, we identified and performed the functional characterization of genes encoding Pmk1 and Mps1MAPKs in *S. versabilis*. After disrupting each of these genes, we established that both SvPmk1 and SvMps1 had profound effects on the vegetative growth and virulence of *S. versabilis.* The vegetative growths of *∆Svpmk1* and *∆Svmps1* mutants were significantly reduced on the PDA, OA and MEA. This result agrees with previous findings in *S. cerevisiae* where the fus3/kss1 MAPK was required for mating, filamentation and invasive growth; and in *M. oryzae* where Pmk1, the ortholog of fus3/kss1, was essential for hyphal growth [19,49]. The two deletion mutants undergo autolysis on PDA and other culture media used with poor development of aerial mycelia. Poor development of aerial mycelia could be the cause, to a great extent, of pycnidia formation defects and subsequent non-conidiation of the fungus. The phenotypic assay showed that the *∆Svmps1* mutant was highly melanized in vitro. The melanization of the mutant is an indication that SvMps1 is a negative regulator of pigmentation in *S. versabilis*. Melanin is reported to protect fungi against adverse conditions and oxidative stress responses [47,50]−. We expected a similar phenomenon for the *∆Svmps1* mutant; however, the *∆Svmps1* mutant was highly melanized, and it was significantly inhibited by reactive oxygen species (ROS), H_2_O_2_ (Figure 5A,B). Therefore, further research is needed to investigate the mystery behind this contradiction.

The Pmk1 pathway in filamentous fungi is involved in pathogenicity [13,16,25,51,52]. In *P. oryzae* and *U. maydis*, Pmk1 is essential for appressorium formation [13,25], an infection structure that aids in the penetration and subsequent invasion of the host cells. Similarly, in non-appressorium-forming fungi of agricultural importance such as *F. graminearum* and *Botrytis cinerea*, Pmk1 and Mps1 are required for penetration and colonization of the host cell [13,51,52]. Previous studies showed that in *Bipolaris oryzae* and *Fusarium Oxysporum*, the loss of Pmk1 abolished pathogenicity; however, the deletion of *MPS1* had no pathogenic or vegetative implication on the two fungi [25]. Similar to these results, our pathogenicity assay with the host leaf indicated that the deletion of *SvPMK1* and *SvMPS1* had a significant adverse effect on the virulence of *S. versabilis.* There was a delay in invasive growth of the hyphae with a restriction to the primary host cell after 1–4 weeks post-inoculation, indicating the pathway’s involvement in the pathogenicity of *S. versabilis*. The mutants failed to cause brown spot lesions beyond the wounded inoculation sites on the host leaves. The pathogenicity defects of *S. versabilis* resulting from the disruption of these critical genes were restored in the complemented strains.

As in many phytopathogenic fungi, hyphal growth and conidiation are crucial for the propagation and disease cycle of *S. versabilis*. When the propagules or spores land on the host surface under favorable conditions, they germinate to form germ tubes that develop into appressorium as in *U. maydis*, or the hyphae may penetrate the host tissue directly without any specialized structure [52]. A reproduction analysis of the two MAPK mutants indicated that SvPmk1 and SvMps1 are essential for sexual reproduction in *S. versabilis*. This result is consistent with what was observed in *Cochliobolus heterostrophus* [25]. Still, it contradicts the finding in *F. verticilloides*, where *Fvmk1* mutant was normal in sexual reproduction and produced macroconidia [52].

Fungal cell walls are a dynamic multipolymeric structure, consisting of glucans, melanin, glycoprotein, chitosan and chitin and glycoproteins and lipids that balance viability, morphogenesis and pathogenesis [53,54,55]. Cell walls are essential for cell morphology, integrity [56], strength and rigidity against the internal turgor pressure (of between 0.2 and 10 MPa) that generates the mechanical force that aids the penetration of vegetative hyphae tips through stiff plant cuticles [53]. A high plastic cell wall interacts with external environmental stress sensors and protects the cell against environmental changes [54,57] and adaptation to heat, ROS and antifungals [44]. They drive the host’s immune response to promote growth and dissemination [58]−. It has been reported that the disruption of the cell wall integrity gene leads to mutants showing a defect in cell wall integrity. Jung and Levin identified about 25 cell wall protein-encoding genes involved in cell wall biogenesis and regulated by Slt2 MAPK [59]. For instance, in *S. cerevisiae*, *∆Slt2* deletion mutants showed hypersensitivity to both ethanol and calcofluor white [60]. In *Cryphonectria parasitica*, *CpSLT2*-null mutant exhibited poor penetration and was hypertensive to cell wall stressors [61]. Similarly, our result is consistent with this finding. We established that the *∆Svmps1* mutant strain cultured on PDA supplemented with chitin-binding agents (CR and CFW) and detergent (SDS) displayed a severe growth defect in response to CR and CFW but not SDS, suggesting a link to cell wall integrity. The *∆Svpmk1* mutant was less inhibited in all three media (except SDS) than the WT and *∆Svmps1*, indicating that SvPmk1 is not required for cell wall integrity, but may contribute to membrane integrity. More so, more protoplasts were released in the *∆Svmps1* mutant than the WT and *∆Svpmk1* after treatment with a cell wall-degrading enzyme. Therefore, the SvMps1 component of the MAPK cascade can be an excellent target for breeding and antifungal formulation.

As with several pathogenic fungi, the cell wall integrity pathway in *S. versabilis* involves reactive oxygen species (ROS) and resistance to fungicides. In host–pathogen interactions, one of the defense responses of host plants to pathogenic attack is the release of ROS as an antimicrobial compound [62,63]. The overproduction of ROS inhibits hyphal growth by causing localized cell death around the site of inoculation [63]. Furthermore, pathogen-induced extracellular ROS secretion is critical for fungal growth and pathogenesis-related development during host–pathogen interaction [64]. However, pathogenic fungi have their techniques of perceiving, detoxifying and tolerating plant-secreted ROS for successful colonization of the host tissue [63]. For instance, the *∆Mohyr1* mutant lacking glutathione peroxidase in *P. oryzae* showed growth inhibition with the increased concentration of H_2_O_2_ [62], indicating the role of MoHyr1 in ROS tolerance. In *P. oryzae*, the loss of MoAtf1 led to hypersensitivity of the *∆Moatf1* mutant to oxidative stressors and reduced extracellular peroxidases and laccases [25]. On the other hand, Rac1 interacts with Nox1 and Nox2 in the signaling pathway to produce ROS required for infection-related development and pathogenicity in *P. oryzae* [7]. In *U. maydis*, *rac1* mutants were impaired in vegetative growth and became non−pathogenic as a result of the low production of ROS [7]. NADPH oxidase 1 (Nox1), suspected to function upstream of the Ste1 MAPK pathway [7], needs to be available at an optimal state to respond to upstream signals [65]. Similarly, our functional characterization result showed that SvMps1 of *S. versabilis* was involved in plant-generated ROS tolerance. The deletion of *SvPMK1* reveals that this gene had no significant effect on ROS formation in *S. versabilis.* However, the growth of the *∆Svmps1* mutant was highly inhibited on PDA media supplemented with increased H_2_O_2_, failed to produce conidia and caused a defect in virulence. This defect could be attributed to the mutant’s inability to sense and detoxify plant-produced ROS in the early stage of infection.

Salts that cause osmotic and ionic stress, such as the toxicity of Na^+^ [66], are environmental factors that affect the growth and proliferation of many living organisms. In *S. cerevisiae*, cds1 and cho1 were identified by Yin et al. [67] to promote tolerance to NaCl-induced stress. Mps1 was reported to be responsible for cellular adaptation to osmotic imbalance in some pathogenic fungi [17]. In this study, we noticed that the rate of mycelial growth inhibition of *∆Svmps1* mutant on PDA containing 1 M sorbitol was about−0%, while that of *∆Svpmk1* was 50%. The high negative value of the inhibition rate of vegetative growth of the *∆Svmps1* mutant suggests that the *SvMPS1* gene is not essential for sensing sorbitol stress in contrast to the *∆Svpmk1* strain that is severely inhibited relative to the WT and *∆Svmps1*. Additionally, this finding agrees with Turrà and co-authors [7] who stated that the restricted colony growth of *slt2* ortholog mutants of *P. oryzae* and *F. graminearum* could be partially restored by sorbitol supplementation. This shows that SvPmk1 and SvMps1 act antagonistically in the MAPK pathway for adaptation to sorbitol stress.

In the *∆Svmps1-Com* strain, the inhibition rate was less than that of the WT. More so, compared to the WT, the growths of *∆Svpmk1* and *∆Svmps1* mutants were less restricted in KCl and NaCl, with *∆Svmps1* attaining almost zero inhibition rate in KCl. In general, we conclude that SvPmk1 and SvMps1 are not important for osmoregulation in *S. versabilis* and are negative regulators of adaptation to salt stress.

In summary, our functional characterization of SvPmk1 and SvMps1 in *S. versabilis* revealed that the two gene products differentially regulate the vegetative and invasive growths, development, reproduction and pathogenicity of *S. versabilis*. Moreover, we established that SvMps1 is required for the maintenance of cell wall integrity and oxidative stress tolerance in *S. versabilis*. Although SvPmk1 is essential for sorbitol sensing, our statistical analysis suggested that SvPmk1 and SvMps1 are not crucial for osmoregulation in *S. versabilis*. These findings provide the molecular bases for unveiling the mechanism of pathogenicity of the fungus.

## Data Availability

No new data were created.

## Supplementary Materials

The following supporting information can be downloaded at: https://www.mdpi.com/article/10.3390/jof9040455/s1, Figure S1: Illustration of target gene knockout strategy. ORF was replaced with Hph encoding for hygromycin resistant gene; Figure S2: Confirmation of *∆Svpmk1* and *∆Svmps1* deletion mutants by PCR and Southern blot analysis; Table S1: Primers and its sequences used in gene knock out.
