# Supplementary material for: Mitogen-Activated Protein Kinases SvPmk1 and SvMps1 Are Critical for Abiotic Stress Resistance, Development and Pathogenesis of Sclerotiophoma versabilis
Source: J Fungi (Basel). 2023 Apr 7;9(4):455. doi: 10.3390/jof9040455 (PMC10142639; doi:10.3390/jof9040455)
Supplement: Supplementary file 1 [file jof-09-00455-s001.zip › Supplementary Figures.pdf]

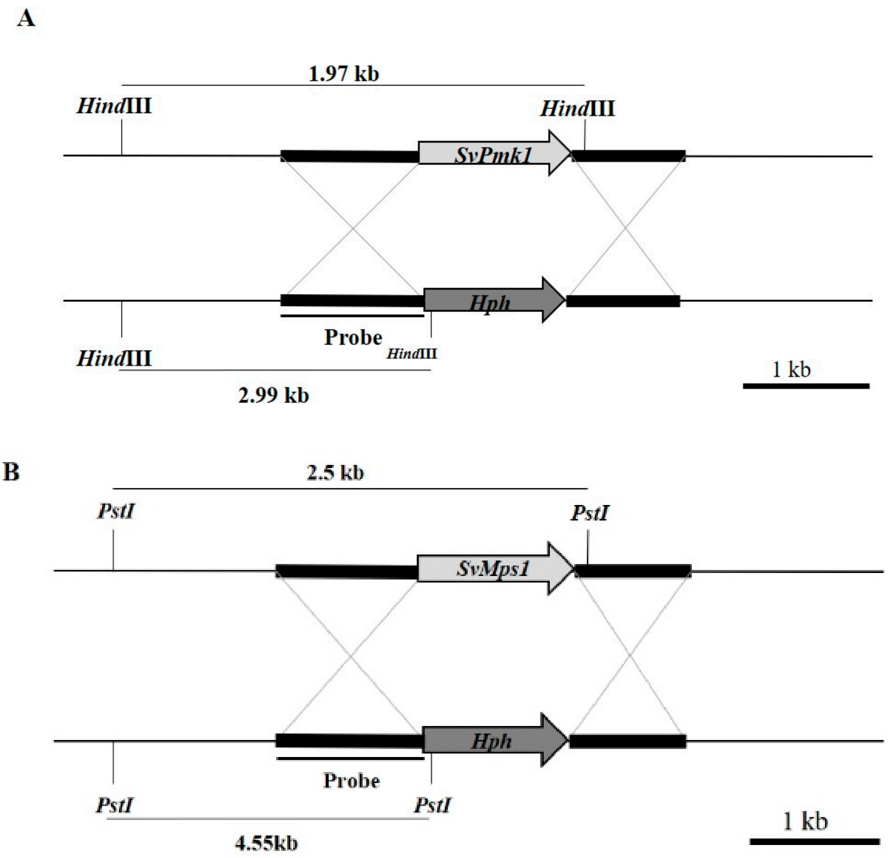

Supplementary Figure S1: Illustration of target gene knockout strategy. ORF was replaced with Hph encoding for hygromycin resistant gene. **A** and **B**. Sketch representation of *SvPmk1* and *SvMps1* deletion in *S. versabilis* genome. The knockout vector was constructed to get the recombination fragments to replace the entire *SvPmk1* and *SvMps1* gene with the hygromycin resistance cassette (HYG). Marker: 3.0 kb.

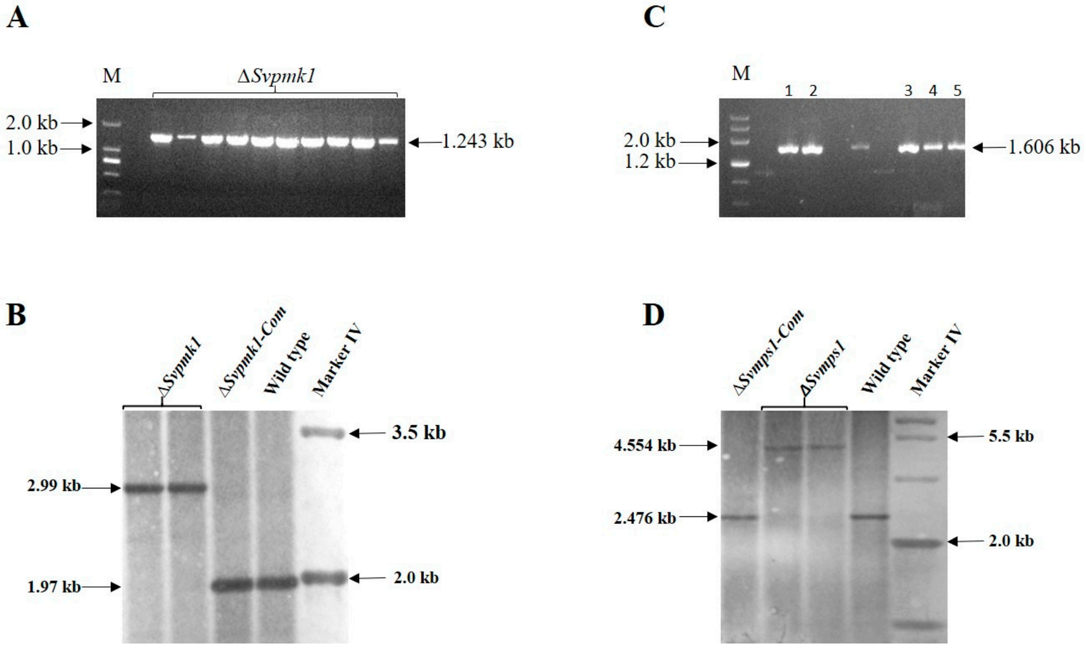

Supplementary Figure S2: Confirmation of Δ*Svpmk1* and Δ*Svmps1* deletion mutants by PCR and Southern blot analysis. **A**. Electrophoretic screening of Δ*Svpmk1* mutants. **B**. Southern blot assay of the Δ*Svpmk1* deletion mutants, WT and complementation using B fragment (downstream) as a probe. **C**. Electrophoretic screening of Δ*Svmps1* mutants. **D**. Southern blot assay of Δ*Svmps1* deletion mutants, WT and complementation strain using A fragment as a probe (upstream). 1 – 5 represents Δ*Svmps1* transformants. M = Marker: IV.
