# Supplementary material for: Mitogen-Activated Protein Kinases SvPmk1 and SvMps1 Are Critical for Abiotic Stress Resistance, Development and Pathogenesis of Sclerotiophoma versabilis
Source: J Fungi (Basel). 2023 Apr 7;9(4):455. doi: 10.3390/jof9040455 (PMC10142639; doi:10.3390/jof9040455)
Supplement: Supplementary file 1 [file jof-09-00455-s001.zip › Supplementary Table.pdf]

Supplementary Table S1: Primers and its sequences used in gene knock out

Primer name	Sequence 5′ - 3′
SvPmk1 – AF	**GAACAAAAGCTGGGT**TCAGACAGAGGTGCTACTTTCGTG
SvPmk – AR	**CAGCGGCGCGCCGAA**TTGGCGGTGTTTGTGGGTT
SvPmk1 – BF	**ACCGGGCCGGCCGGA**TTTTGACGGTAGTAGGAACGAATAAG
SvPmk1 – BR	**GGTGGCGGCCGCTCT**GAGAAGGATGCTGAACAGAGGC
SvPmk1 – OF	AAGATCACTCCGTTCGACCACT
SvPmk1 – OR	CAGCCAAGATACAGCCAACACTC
SvPmk1 – UF	TCACTGCCACAACTGCTTC
SvPmk1 – UR	GGGCGTCGGTTTCCACTA
SvPmk1 – ComF	**GAACAAAAGCTGGGT**TGTTGACGTAGTCGCATATC
SvPmk1 – ComR	**CTGCAGGCATGCAAG**TCGCATGATCTCCTGGTAGA
SvMps1 – AF	**GAACAAAAGCTGGGT**TGTTTCACCGATTTACGC
SvMps1 – AR	**CAGCGGCGCGCCGAA**TGAAGCACCACTCAGCAT
SvMps1 – BF	**ACCGGGCCGGCCGGA**CCTTGAACCTACCAACCCTA
SvMps1 – BR	**GGTGGCGGCCGCTCT**CAGATACGCCGTTACCCT
SvMps1 – OF	CCCCGAACATCGCTGACA
SvMps1 – OR	GGTTGGCGTTGCGGAAGA
SvMps1 – UF	TGATGGTGCGAGAAAAGA
SvMps1 – UR	TGAAATAAAGGGAGGAAGG
SvMps1 – ComF	**GAACAAAAGCTGGGT**TCTCCATTGTTTCACCGATTTACGC
SvMps1 – ComR	**CTGCAGGCATGCAAG**TCTCATCCGGCCGTCCAAGC
YG – F	GATGTAGGAGGGCGTGGATATGTCCT
HY – R	GTATTGACCGATTCCTTGCGGTCCGAA
HYG/F	GGCTTGGCTGGAGCTAGTGGAGGTCAA
HYG/R	AACCCGCGGTCGGCATCTACTCTATTC
PKNTG-GFP-F	CACAAGTTCAGCGTGTCCG
PKNTG-GFP-R	GTTCACCTTGATGCCGTTC

Note: Bold represents restriction enzyme sites.
